# Involvement of Tumor Macrophage HIFs in Chemotherapy Effectiveness: Mathematical Modeling of Oxygen, pH, and Glutathione

**DOI:** 10.1371/journal.pone.0107511

**Published:** 2014-10-08

**Authors:** Duan Chen, Andrey A. Bobko, Amy C. Gross, Randall Evans, Clay B. Marsh, Valery V. Khramtsov, Timothy D. Eubank, Avner Friedman

**Affiliations:** 1 Department of Mathematics and Statistics, University of North Carolina at Charlotte, Charlotte, North Carolina, United States of America; 2 Division of Pulmonary, Allergy, Critical Care and Sleep Medicine, College of Medicine, The Ohio State University, Columbus, Ohio, United States of America; 3 Mathematical Biosciences Institute, The Ohio State University, Columbus, Ohio, United States of America; 4 Department of Mathematics, The Ohio State University, Columbus, Ohio, United States of America; University of Pittsburgh, United States of America

## Abstract

The four variables, hypoxia, acidity, high glutathione (GSH) concentration and fast reducing rate (redox) are distinct and varied characteristics of solid tumors compared to normal tissue. These parameters are among the most significant factors underlying the metabolism and physiology of solid tumors, regardless of their type or origin. Low oxygen tension contributes to both inhibition of cancer cell proliferation and therapeutic resistance of tumors; low extracellular pH, the reverse of normal cells, mainly enhances tumor invasion; and dysregulated GSH and redox potential within cancer cells favor their proliferation. In fact, cancer cells under these microenvironmental conditions appreciably alter tumor response to cytotoxic anti-cancer treatments. Recent experiments measured the *in vivo* longitudinal data of these four parameters with tumor development and the corresponding presence and absence of tumor macrophage HIF-1*α* or HIF-2*α* in a mouse model of breast cancer. In the current paper, we present a mathematical model-based system of (ordinary and partial) differential equations to monitor tumor growth and susceptibility to standard chemotherapy with oxygen level, pH, and intracellular GSH concentration. We first show that our model simulations agree with the corresponding experiments, and then we use our model to suggest treatments of tumors by altering these four parameters in tumor microenvironment. For example, the model qualitatively predicts that GSH depletion can raise the level of reactive oxygen species (ROS) above a toxic threshold and result in inhibition of tumor growth.

## Introduction

Tumors have distinguishing features from normal tissue. Among the most significant factors in tumor metabolism and physiology are the tissue oxygen concentration, acidity, intracellular glutathione (GSH) concentration and redox status [1]–[5]; in the sequel we focus on the first three features.

### (i) Tissue oxygen level

Clinical investigation has shown that hypoxic regions develop in a wide range of malignancies including cancers of the breast, uterine cervix, and prostate. Inefficient tumor vasculature induces hypoxia which decreases extracellular pH and increases interstitial fluid pressure. Hypoxia-induced transcription factors like HIF-1*α* regulate VEGF and other glucose-regulating genes like GLUT-1 which augments glucose uptake from the surroundings. This process favors tumor cell proliferation as tumor cells generate 50% of their ATP from glycolysis while normal cells generate only 10%, giving tumor cells an adaptive survival advantage over adjacent normal cells. Further, tumor hypoxia is associated with poor patient prognosis because low oxygen reduces the effectiveness of therapies that require the generation of ROS for cell killing.

### (ii) Tumor acidity

Tumor acidity is due to increased lactic acid secretion from the anaerobic metabolism of cancer cells via their expression of tumor M2-PK, a dimeric isoenzyme of pyruvate kinase up-regulated in cancer cells. M2-PK drives pyruvate to lactate, a major energy source in tumors [6]. In turn, tumors have a lower extracellular pH (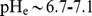) [2], [7] maintained by increased carbonic anhydrase IX(CAIX) activity compared to normal tissue (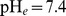) [8], [9]. Extracellular acidity results in increased tumor invasion, proliferation, evasion of apoptosis, and cell migration as well as ion trapping of weak base drugs [2]. A sequence of interdisciplinary studies, involving mathematical models and experimental evidences, have been conducted in [10]–[12], for the tumor-stromal interactions and acid-mediated tumor invasion. More recently, the anti-cancer effects of pH buffer therapy was investigated in [13] and variety of foods were suggested that can contribute to manage cancers.

### (iii) Intracellular glutathione

GSH plays a crucial role in balancing redox status in tumor microenvironment [14], [15]. Indeed, accumulated evidence indicates that increased level of hydrogen peroxide (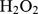) and other reactive oxygen species (ROS) occur in many types of cancer cells compared to their normal counterparts through far greater rates of mitochondrial reduction of superoxide [16]. Within a certain range, increases in ROS promote tumor cell proliferation by activating glucose-regulating genes and production of angiogenesis signaling factors like VEGF, whereas ROS leads to oxidative damage (ROS stress) at levels above a toxicity threshold. As a major intracellular redox buffer and antioxidant for redox adaption [17] and in response to ROS stress, high levels of GSH have been found in various tumor types, being up to several-fold greater than that in surrounding tissues [18].

In the present paper we develop a mathematical model for tumor growth with dynamics of GSH concentration, pH and oxygen tension in the tumor microenvironment. This is a two-scale model: at the tissue level, the interactions between tumor, immune, and endothelial cells, along with corresponding cytokines, are modeled by a set of partial differential equations (PDEs) in a moving domain, in which a velocity field is included to describe the movement of cells, chemicals, and the tumor boundary; at the cellular level, a dynamical system of intracellular chemical interactions between ROS, GSH, and other intermediate molecules is proposed within individual cells. We validate the model by comparing simulations to experimental data, and then use the model to predict tumor growth with intracellular GSH depletion as a possible therapeutic strategy. The model can also be used to monitor the change of pH, GSH and oxygen in tumor as a result of the absence or presence of macrophage HIF*α*s (HIF-1*α* or HIF-2*α*) and corresponding effectiveness of chemotherapeutic drugs. The footprint of these quantities could relate to the efficiency of a drug in terms of the tumor microenvironment. We illustrate this approach by simulation of the course of tumors treated by docetaxel (DTX).

## Mathematical Model

In this section we describe a mathematical model representing tumor growth along with dynamics of GSH concentration, pH and oxygen level, by a system of ordinary and partial differential equations. At the tissue level, we have cancer cells interacting with immune cells and the vascular system during angiogenesis, while at the cellular level we have GSH, pH and oxygen concentrations interacting within each cancer cell.

### Variables and relations

For tumor growth at the *macroscopic* scale, we have as variables the densities of live and dead tumor cells, macrophages, and endothelial cells (ECs), and the concentrations of cytokines interacting among the cells: monocyte chemoattractant protein-1 (MCP-1/CCL2), vascular endothelial growth factor (VEGF), and soluble VEGF receptor-1 (sVEGFR-1). Two other macroscopic variables are oxygen tension and concentration of hydrogen ions, which can be measured experimentally. At the *microscopic* level, we consider the intracellular concentrations of ROS, GSH, and reduced/oxidized forms of GSH peroxidase (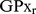/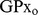). A list of all these variables is given in Table 1.

**Table 1:** Variables and units of the model.

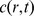	live tumor cell density (cell/ 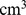 )
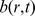	dead tumor cell density (cell/ 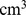 )
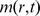	macrophage density (cell/ 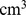 )
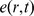	endothelial cell density (cell/ 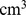 )
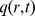	M-CSF concentration (g/ 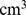 )
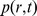	MCP-1/CCL2 concentration (g/ 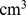 )
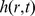	VEGF concentration (g/ 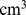 )
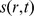	sVEGFR-1 concentration (g/ 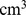 )
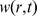	Oxygen concentration (g/ 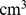 )
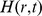	Concentration of 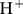 ( 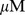 )
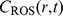	Concentration of ROS (mostly 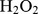 ) ( 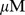 )
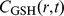	Concentration of GSH ( 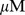 )
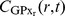	Concentration of 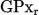 ( 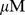 )
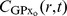	Concentration of 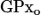 ( 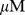 )

**Figure 1:**
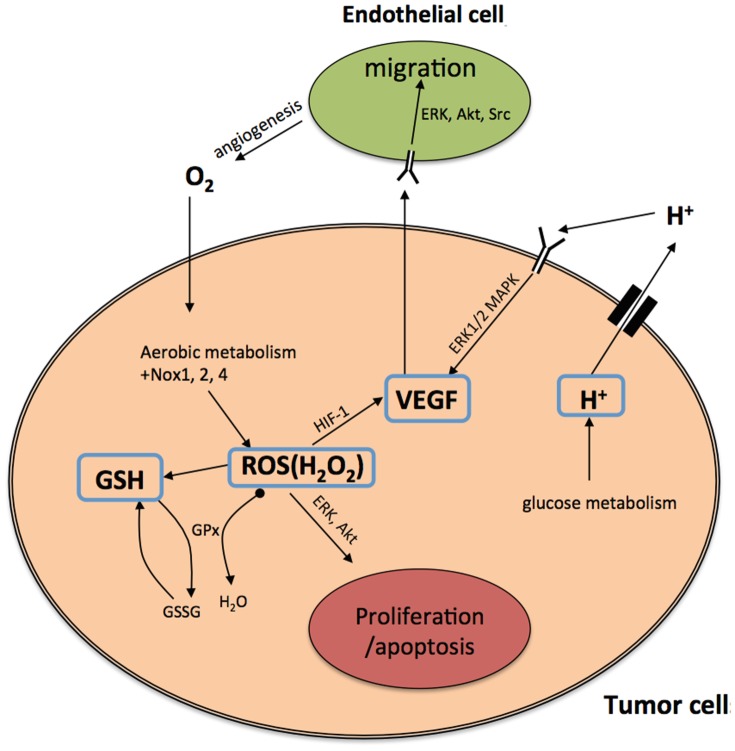
Schematic diagram of the roles of ROS, GSH, and hydrogen ions in cancer cell growth and tumor angiogenesis. (i) ROS is a major by-product of aerobic metabolism and plays a dual role in cancer cell life-cycle: below a certain threshold, increasing amounts of ROS promotes cell proliferation through pathways of extracellular-signal-regulated kinases (ERKs) and cell survival factors such as Akt. However, ROS leads to cell apoptosis when its concentration is over the toxic threshold. Additionally, ROS may play a function in up-regulating HIF-1 expression, which in turn results in increasing the production of angiogenesis factor VEGF. (ii) GSH (glutathione) is the most abundant antioxidant produced by cancer cells to protect themselves from oxidative stress; it can remove ROS (mostly 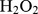) with the help of enzyme 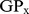. (iii) Large amount of hydrogen ions are produced as a consequence of glucose metabolism, and are pumped out by abnormally expressed proton transporters. There is evidence indicating that acidic extracellular environment induces VEGF production through the ERK/MAPK signaling pathway.

Relations between macroscopic and microscopic variables are described schematically in Figure 1. ROS (primarily 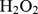) is an important by-product of aerobic metabolism and plays the role of a double-edged sword [15] in cells: when below a certain toxicity threshold 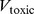, a moderate increase in ROS level could promote cell proliferation, but when it is increased above the threshold, the elevated ROS concentration will trigger cell death. GSH is the most abundant antioxidant produced by cancer cells to protect themselves from oxidative stress with the help of the enzyme glutathione peroxidase. On the other hand, large amount of hydrogen ions are produced from glucose or anaerobic metabolism. Low intracellular pH (pH*_i_*) can mediate apoptosis of cancer cells, but the access protons are pumped out by over-expressed proton transporters [19], and this leads to an acidic extracellular environment (low 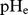). Indeed, the experimental measurements in the current work are about 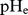 and thus only the extracellular acidosis-induced release of VEGF mentioned in [10] is considered. It was illustrated in [20] that VEGF promoter activity is inversely correlated with tumor extracellular pH *in vivo* in the human glioma xenografts. Additionally, it was concluded in [21], [22] that below the toxic threshold, ROS also contributes to upregulation of HIF-1*α* protein expression, which further enhances VEGF expression. Therefore, the levels of pH and ROS are linked to angiogenesis through VEGF production.

### Macroscopic tumor growth model

**Figure 2:**
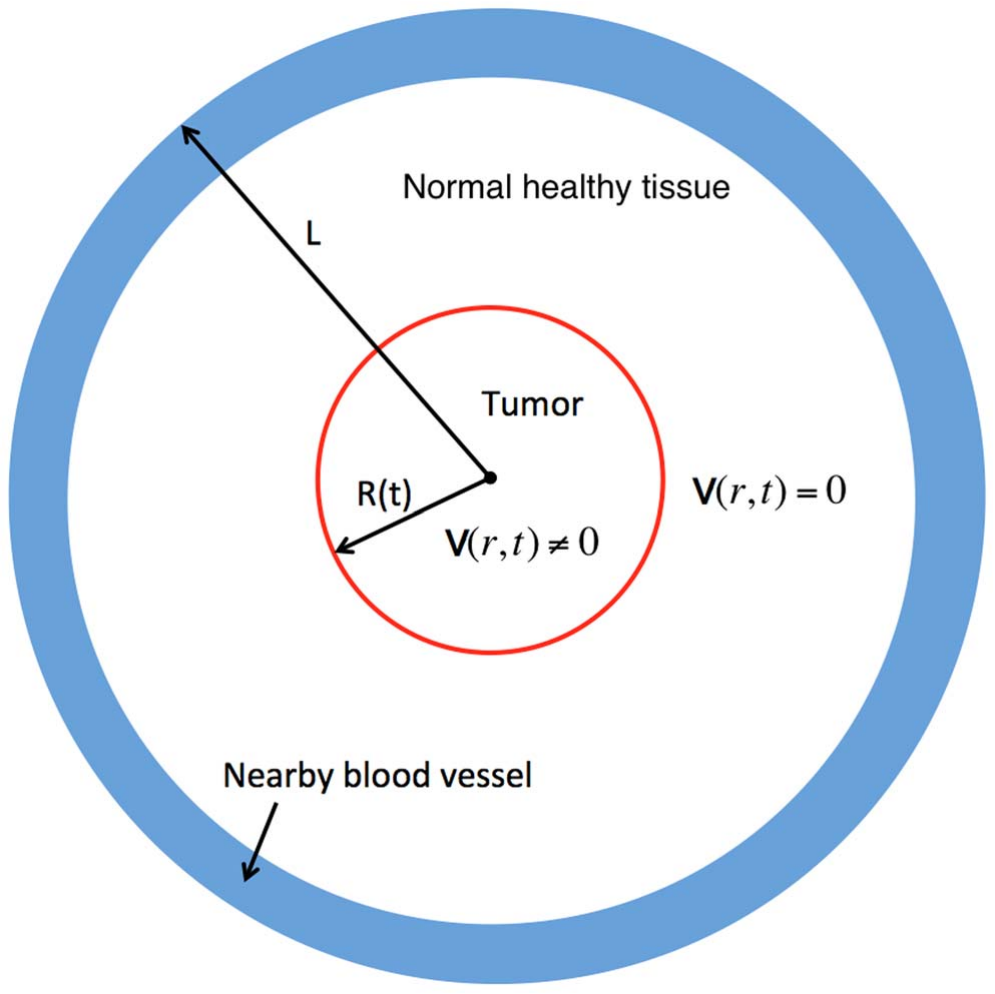
Macroscopic tumor growth: tumor is assumed to be in radially symmetric with radius 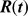 evolving in time *t*; the moving boundary is indicated in red. A healthy normal tissue surrounds the tumor, and the entire simulation domain is a sphere with a fixed radius *L*. Initially, blood vessel (in blue) is placed in the healthy normal tissue away from the tumor region. Due to abnormal proliferation of cancer cells, there is a radial velocity, 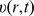, within the tumor region, but 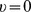 in the healthy normal tissue.

For model simplicity, the tumor is assumed to be a sphere with radius 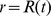 evolving in time (see Fig. 2), which is embedded in a larger sphere with a fixed radius 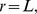 whose boundary lies in a normal healthy tissue. The proliferation of tumor cells generates an internal pressure and, as a result, a velocity field with radial velocity 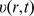 outward from the center. We assume that all cells and molecules are moving with this velocity; the velocity is zero in the normal tissue. The equations for live and dead tumor cells are defined in the moving domain 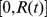 whereas equations for all other cells and chemicals take place in the fixed domain 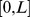.

Equations of the macroscopic variables are based on the framework in [23] with some changes due to intracellular reactions. The equation of live cancer cells is:
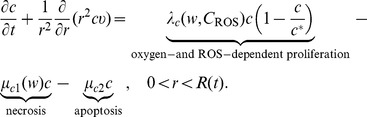
(1)


Here, the proliferation rate 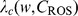 depends on the oxygen level 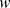 and intracellular ROS concentration, 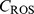; we assume that 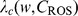 has the form
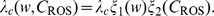



**Figure 3:**
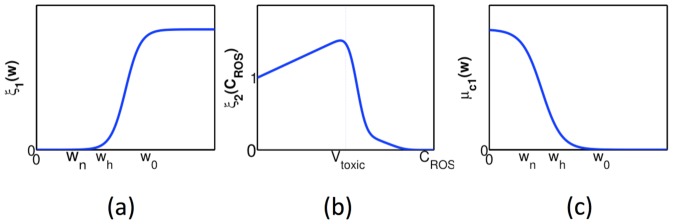
Profiles of the functions 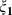, 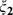, and 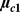. Thresholds of necroxia, hypoxia and normoxia are marked as 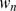, 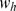 and 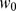, respectively.

We also assume that the necrosis rate 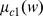 is only oxygen-dependent and the apoptosis rate 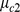 is a constant. The forms of the functions of 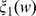, 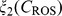, and 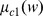 should have the profiles shown in Fig. 3, but for numerical simulation we approximate them by piecewise linear functions.

The functions 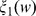 and 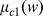 are taken the same as in [23],
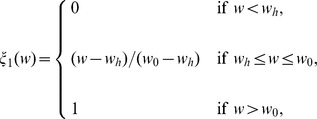


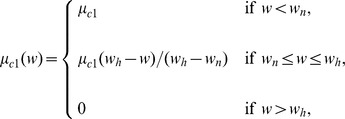
where 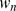, 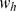 and 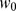 represent thresholds of necroxia, hypoxia and normoxia, respectively.

In the experiments in ovarian cancer in [24], tumor volume was almost doubled when the intracellular ROS level was elevated by 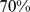, so we estimate by 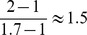, the fold at which ROS level increases proliferation of cancer cells. Accordingly, we take the function 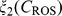 as follows:
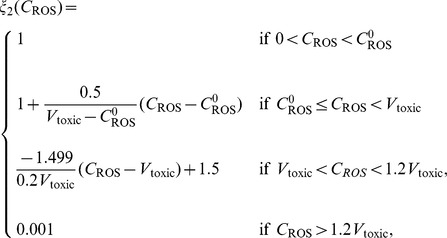
where 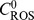 is the typical ROS concentration in cancer cells.

The VEGF density satisfies the equation
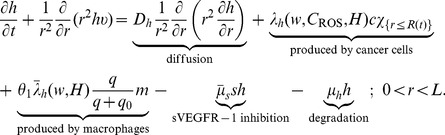
(2)


In this equation, the diffusion coefficient 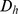, binding rate 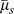 to sVEGFR-1, and degradation rate 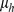 are assumed to be constant. The parameter 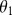 is set as one for normal macrophages but zero for HIF-1*α*-deficient macrophages [23]. Here, we assume that the VEGF production rate has the form: 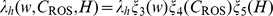. We take
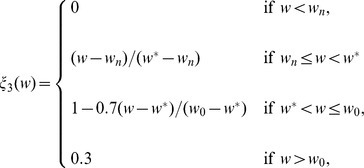
where 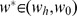 represents the threshold at which the hypoxic effect is maximal for VEGF production [23].

Up to five-fold increase of the maximum HIF-1*α* expression was suggested by a cancer model in [25] when the ROS level was elevated. Hence we take the function 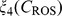 to be similar to 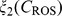:
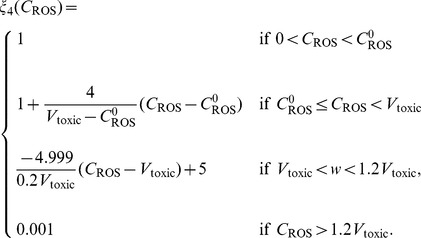



The VEGF promoter activity at extracellular pH = 6.6 is three-fold higher than that at pH = 7.3 [7]. In [26], the pH values are 7 and 6.55 for mammary gland and non-treated MET-1 tumor, which correspond to 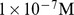 and 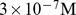 of hydrogen ion concentrations, respectively. Accordingly we set 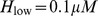, 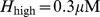, and take
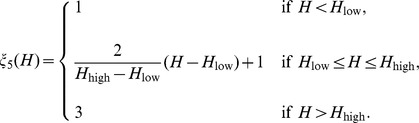



Finally, for simplicity, we set 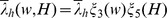 [23].

The equation for the concentration of hydrogen ions is given by
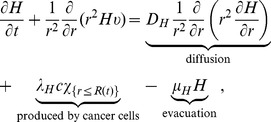
(3)where 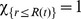 if 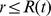 and 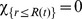 if 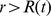, and where 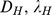 and 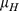 are the diffusion coefficient, production and evacuation rate of hydrogen ions, respectively; for simplicity, they are assumed to be constant.

The equations for oxygen level 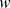 and density of macrophages 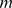 appearing in Eqs. (1) – (2), and of the other variables in Table 1 are the same as in [23], except for additional terms involving 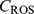 and 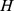.

Collecting the equations for all the macroscopic variables listed in Table 1, we have the following system:
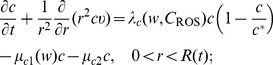
(4)

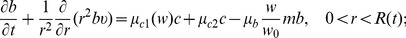
(5)

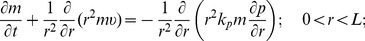
(6)

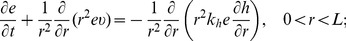
(7)

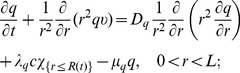
(8)

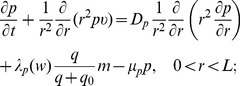
(9)

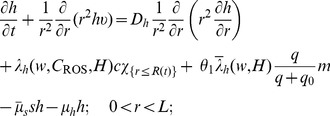
(10)

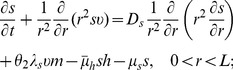
(11)

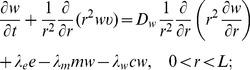
(12)

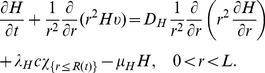
(13)


The radial velocity field 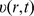 and the moving boundary 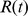 of the tumor are given by
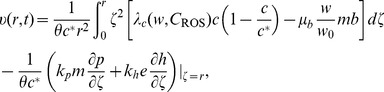
(14)where 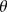 is the fraction of the volume occupied by cells, and



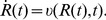
(15)Detailed discussions about the corresponding terms and coefficients of Eqs. (5) – (9), (11) – (12) and derivations of (14) – (15) can be found in [23], but for convenience, all the parameters are listed in Table 2.

**Table 2:** Values and reference of parameters in the macroscopic equations (4) – (15).

Parameter	Dimensional	Reference
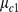	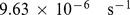	[57], [58] and estimated
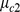	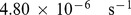	[59]
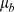	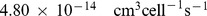	[59] and estimated
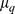	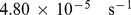	[60]
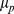	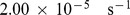	[61]
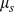	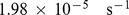	[62]
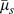	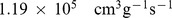	[63] and estinated
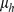	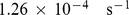	[64]
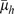	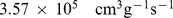	[63] and estimated
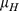	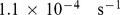	[29]
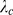	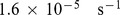	[57], [58]
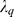	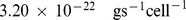	[65], [66] and estimated
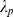	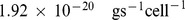	[67] and estimated
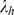	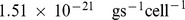	[57], [61], [68], [69] and estimated
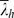	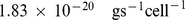	[70], [71] and estimated
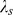	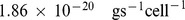	[72] and estimated
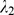	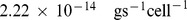	estimated
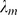	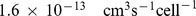	[73] and estimated
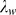	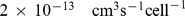	[74], [75] and estimated
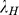	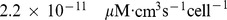	[29]
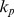	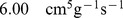	[57]
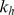	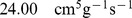	[76]
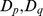	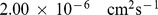	[57], [61], [77] and estimated
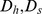	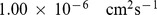	[76], [78] and estimated
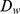	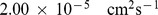	[79] and estimated
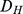	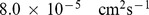	[30], [31]
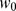	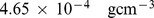	[80]
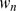	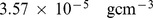	[80] and estimated
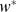	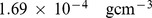	estimated
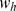	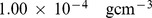	[80] and estimated
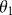	0 for HIF-1*α* KO, otherwise 1	estimated
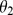	0 for HIF-2*α* KO, otherwise 1	estimated
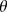	0.9	[23], estimated
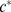	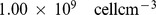	[81]
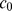	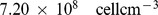	[81], [82]
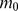	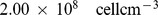	[81]
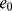	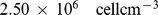	estimated
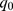	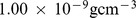	Scaling factor
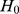	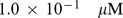	[26]

### Intracellular chemical dynamics

As mentioned earlier, 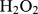 is the major source of ROS. Recent experimental data indicate that an increase of 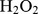 can explain many hallmarks of cancer, such as cell proliferation, apoptosis resistance, increased angiogenesis, and metastasis [27]. We assume that ROS concentration (which is primarily 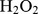) is mainly regulated by GSH, although there exist other reducing agents [1]. Removal of 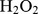 by GSH is associated with a key enzyme, glutathione peroxidase (GPx). In fact, the reactions of intracellular 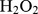, GPx and GSH are [28]:
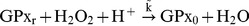
(16)

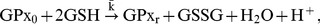
(17)where 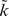 and 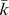 are reaction constants, and 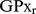 and 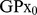 are the reduced and oxidative forms of 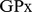, respectively.

Based on (16) and (17), the dynamical system for 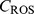, 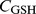, 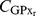 and 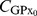 are modeled as the follows:
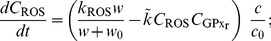
(18)

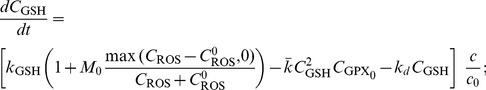
(19)

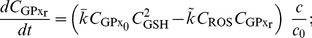
(20)

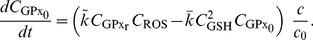
(21)


The equations of 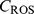, 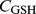 and concentrations of the other associated molecules take place inside cancer cells. In Eq. (18), ROS is produced at the oxygen level-dependent rate 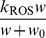 and is removed by 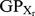 as indicated by Eq. (16). For Eq. (19), GSH is generated inside cancer cells, at a constant rate 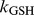. When cells are under oxidative stress (ROS concentration is above normal level 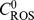, or 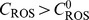), they acquire adaptive mechanisms to counteract the toxicity of increased ROS level by upregulating GSH synthesis. Hence, a 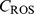-dependent GSH production is included with 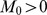. A phenomenological value 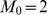 is taken in this model since there is no experimental evidence, to the authors' knowledge, about how much GSH production is enhanced due to oxidative stress. Further, GSH is consumed by 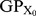 as indicated by Eq. (17), and degrades at a constant rate 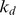. Eqs. (20) and (21) are directly derived from reactions (16) and (17). Since these reactions take place inside cancer cells, in order to couple the ODE system to the macroscopic tumor growth model, all the right-hand sides of Eqs. (18) – (21) are multiplied by 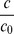, where 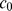 is the reference density of cancer cells.

### Initial and boundary conditions

Initial and boundary conditions corresponding to Eqs. (4) – (12) follow those in [23]:
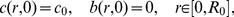
(22)

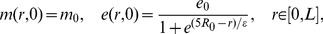
(23)

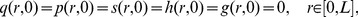
(24)

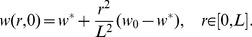
(25)


At 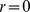, we have zero flux boundary conditions for 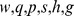, and at 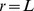, we impose zero flux boundary conditions for 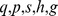, except for 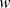, and 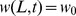.

Next, for 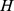, we take the initial condition 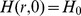, and the boundary condition 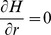 at 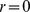 and 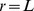. For Eqs. (18) – (21), initial conditions are taken as
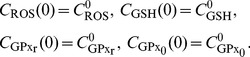
(26)


## Parameters

### Parameters in tumor growth

Values of parameters 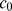, 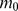, 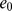, 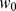, 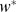, and 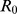 are taken the same as in [23] and are listed in Table 2. Additionally, in [29], tumor interstitial pH profiles in normal and neoplastic tissue were measured *in vivo* by a fluorescence ratio imaging microscopy technique. Based on the experimental data, it was concluded in [29] that the production rate of 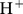 ranges from 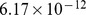 to 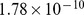 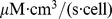, and evacuation rate ranges from 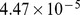 to 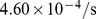. We take the geometric means of these observations and set 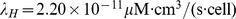 and 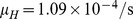. The diffusion coefficient of protons is generally much larger than those of ions and chemicals; we take 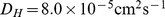, as in [30], [31]. Neutral environment is assumed initially, so 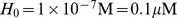 [26].

### Parameters in the intracellular dynamics

It was reported in [32] that for seven adherent human tumor cell lines, including colon and breast cancers, the production of hydrogen peroxide is in the range from 0.1 to 1.4 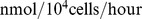. In a more recent work [33], the superoxide production was measured as 3.71 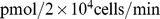 for mouse colon carcinoma and 1.21 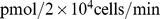 for liver hepatoma. We follow the result in [33] and take the ROS production to be in the range of 1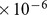 to 3.8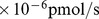 for a single tumor cell, after unit conversion. Assuming the typical volume of a cell to be 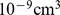, we derive the intracellular ROS production rate 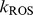 to be
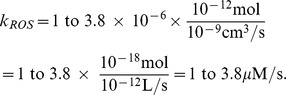



In studies of rat liver mitochondria [28], [34], it was reported that the reaction constants of Eqs. (16) – (17) are 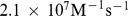 and 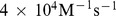, respectively. Thus, after unit conversion we have 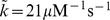 and 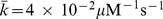.

Glutathione synthesis in red blood cells has been measured in [35] and the production rates are 0.5 mmol/L/day and 1.6 mmol/L/day for young and elderly people, respectively. So we take the constant 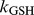 to be
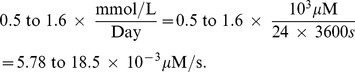



Finally, the degradation rate 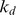 of GSH is in the range from 3.2 to 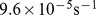 since the half-life of the GSH is between 2 to 6 hours [36], [37]. All the parameter values of Eq. (18) – (21) are summarized in Table 3.

**Table 3:** Values and reference of parameters in the intracellular dynamics of Eqs. (18) – (21).

Parameter	value and unit	Reference
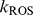	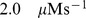	[32]
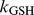	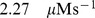	[35], estimated
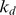	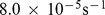	[36], [37]
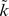	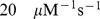	[34]
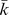	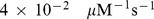	[34]
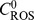	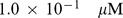	[25], [28], [38]
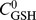	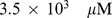	[26]
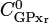	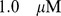	[34], [41], estimated
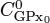	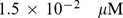	[34], [41], estimated
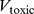	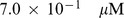	[39], [40]

### Initial conditions

In [28], [38], concentrations of 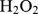 in rat liver cells were found to range from 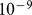
M to 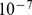
M, and a base value of 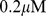 in tumor cells is estimated from the experiments in [25]. Thus, we take 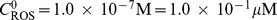; this value is also used as the initial condition of 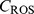. An upper limit of 700 nM for intracellular levels of 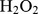 in Jurkat T-cells was suggested in [39], [40], beyond which apoptosis was introduced; hence we take the toxicity threshold of ROS in our model to be 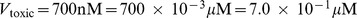. In the experiments of [26], the average intracellular GSH concentration in mammary gland was 3.3 mM, while in tumor it was 10.7 mM; accordingly we take the initial condition of 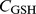 to be 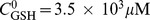. Cellular concentration of 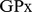 varies from 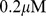 to 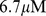 in red blood cells and in other cells [34], [41], and over 99% of it is in reduced form [41], so we take the corresponding initial conditions to be 
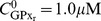
 and 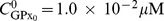.

## Results and Discussion

In this section we present model simulations and compare our results with experimental data. All the simulations were carried out with MATLAB (version R2011a Mathworks). The PDEs of parabolic type were numerically solved using package pdepe (MATLAB function for initial-boundary value problems for parabolic-elliptic PDEs in 1D), and the equations of hyperbolic type were solved by the Semi-Lagrangian scheme. The intracellular dynamics were solved by the ODE solver ode15s.

### Experimental details

#### Mice

6–8 week old C57Bl/6 female mice expressing lysozyme M from the promoter of cre recombinase (LysMcre) were used as wild type control mice. 6–8 week old C57Bl/6 female LysMcre mice also containing homozygous loxP restriction sites surrounding the HIF-1*α* (LysMcre/HIF-1*α* ^fl*/*fl^) or HIF-2*α* (LysMcre/HIF-2*α* ^fl/fl^) genes were used as the experimental groups lacking either HIF-1*α* or HIF-2*α* in the myeloid cells.

#### Tumor model

Met-1 tumor cells isolated from the stage IV tumors of C57Bl/6 PyMT transgenic mice were cultured to 80% confluence then trypsinized, washed, resuspended in RPMI-1640 at 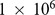 cells per 100 µls and orthotopically implanted into the number four mammary gland of 6–8 week old C57Bl/6 female LysMcre, LysMcre/HIF-1α^fl/fl^, or LysMcre/HIF-2α^fl/fl^ mice. The tumors became palpable approximately 1 week after implantation. Tumor measures were performed 3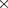 per week using calipers and tumor volumes were calculated using the formula volume  = 0.5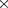 [(large diameter)
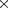
(small diameter)^2^].

#### Treatment

Upon tumor palpation, the mice were treated intraperitoneally with 100 µL isotonic saline or Docetaxel (NDC 0409-0201-02, Hospira) 30 mg/kg body weight in 100 µL one time per week. All protocols were approved by The Ohio State University Animal Care and Use Committee, and mice were treated in accordance with institutional guidelines for animal care.

### HIF-1*α*-regulated tumor microenvironment change

**Figure 4:**
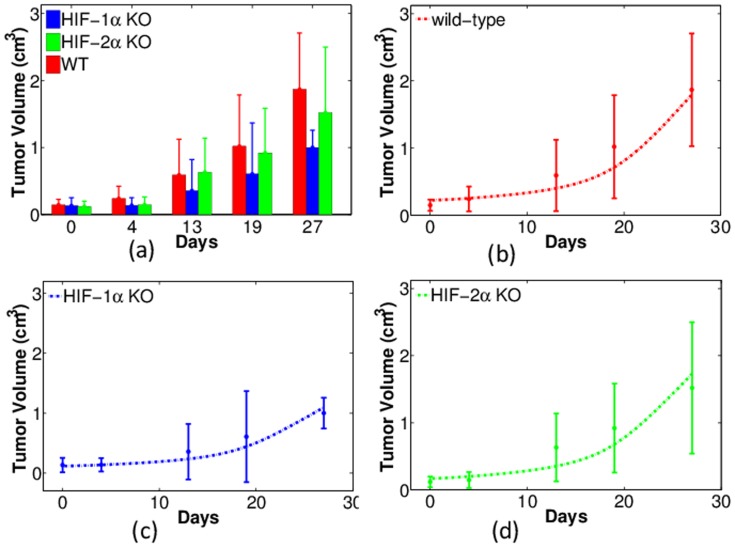
Experiments and simulations of tumor volume with wild-type, HIF-1*α*- and HIF-2*α*-deficient macrophages (WT, HIF-1*α* KO, and HIF-2*α* KO). Horizontal axis represents time (in days) and vertical axis scales tumor volume (in units of cm^3^). (a): Experimental data of tumor volumes with error bars (standard deviations). Red: WT; Blue: HIF-1*α* KO; Green: HIF-2*α* KO. (b)-(d): Comparison of experiments (dots with error bars) and numerical simulations (dash curves) for tumor volumes with WT, HIF1-*α*, and HIF-2*α* KO macrophages, respectively.

Figure 4 shows the comparison between experiments and simulations for tumor volume (in unit of cm^3^) changing with time (days). Fig. 4 (a) lists the experimental data in colored columns with error bars. Unless otherwise specified, the red, blue, and green colors represent tumors with wild-type, HIF-1*α*-, and HIF-2*α*-deficient macrophages (WT, HIF-1*α* KO, and HIF-2*α* KO), respectively. For each type in the longitudinal data, fifteen tumor volumes were measured on each day. The statistical mean of these tumor volumes are calculated and plotted as the heights of the columns, with the error bars as standard deviations. We see that tumors with HIF-1*α* KO macrophages have volumes as low as one half of those with WT macrophages. By contrast, tumor growth is not inhibited if HIF-2*α* in macrophages is knocked out. This agrees with our earlier work about the opposing roles of HIF-1*α* and HIF-2*α* in mediating tumor angiogenesis [23], [42].

In Figs. 4(b) – (d) we compare the model simulations of tumor volume with experiments. In these figures, experimental data are the same as in Fig. 4(a), and are displayed as dots with error bars. For comparison, simulations are plotted in the corresponding colored dash curves. Based on the parameter sensitivity analysis in [23], the parameters 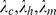 and 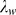 are adjusted to obtain the curves in Fig. 4(b) to fit the experiments; specifically, 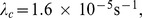 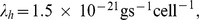 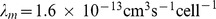, and 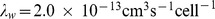. Fixing these parameters but setting 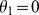 or 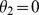 in Eqs. (10) – (11), we obtained the simulations for tumor growth with HIF-1*α* or HIF-2*α*-deficient macrophages and displayed them in Figs. 4(c) – (d), respectively. The agreement of the numerical simulations with experiments is fairly good. The R squared score [43], [44] is used to quantify the goodness of fit; the values for the cases of WT, HIF-1*α* KO, and HIF-2*α* KO are 0.9630, 0.9184, and 0.8917, respectively. Based on the comparison, we proceed to use the model with the same parameters to calculate other quantities in the tumor microenvironment.

**Figure 5:**
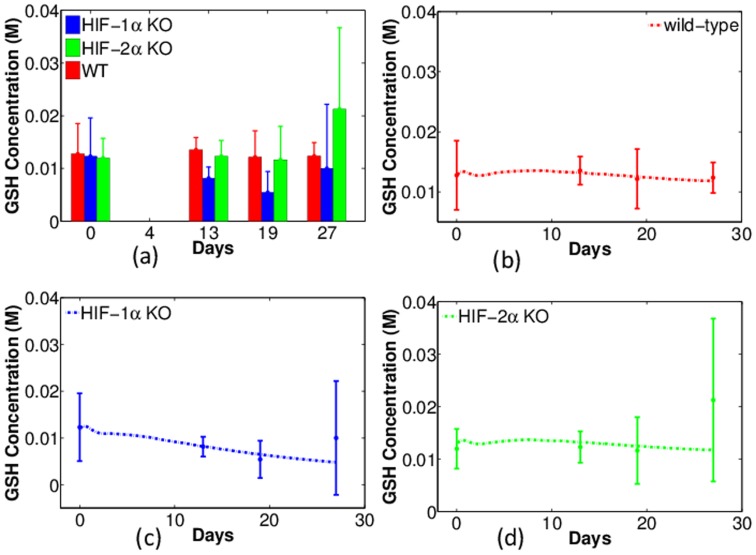
Experiments and simulations of intracellular GSH concentration ([GSH]) in tumors with wild-type, HIF-1*α*- and HIF-2*α*-deficient macrophages (WT, HIF-1*α* KO, and HIF-2*α* KO). Horizontal axis represents time (in days) and vertical axis scales [GSH] in units of Molar. (a): Experimental data of [GSH] with error bars. Red: WT; Blue: HIF-1*α* KO; Green: HIF-2*α* KO. (b) – (d): Comparison of experiments (dots with error bars) and numerical simulations (dash curves) of [GSH] for tumors with WT, HIF-1*α*, and HIF-2*α* KO macrophages, respectively.

Intracellular GSH concentration in normal tissues, non-treated tumors, and GM-CSF treated tumors were explored in [26]. It was concluded that GSH concentration in cancer cells is significantly higher compared with that in normal tissues, and it is lowered when tumor growth is suppressed by GM-CSF treatment. Therefore, based on the previous conclusion that HIF-1*α* KO inhibits tumor growth, we hypothesized that the GSH concentration in tumors with HIF-1*α* KO macrophages is lower than that in tumors with WT or HIF-2*α* KO macrophages. This hypothesis was verified by both experiments and simulations. Figure 5 (a) displays the experiments of GSH concentration (in unit of Molar) against time (days). Similarly, the column heights represent the mean values of the GSH concentration in tumors and the error bars are standard deviations. Note that only one of the quantities (GSH, oxygen and pH) can be measured on each tumor, so that the total number of data point is five per day. From the figure we can see that GSH concentration in tumors with HIF-1*α* KO macrophages (blue bars) is significantly lower, whereas tumors with WT (red) and HIF-2*α* KO macrophages (green) have similar and higher level of GSH concentration in general, except for the measurement on the last day.

Figs. 5(b) – (d) show the simulations corresponding to the three groups of experiments in Fig. 5(a). Since the total sample size is relatively small, the R squared is not calculated. In these simulations, initial average GSH concentration is 0.0125 M. In tumors with WT macrophages, there is no significant change in GSH concentration and after 30 days it is 0.0118 M. A similar pattern is observed in the tumor with HIF-2*α* KO macrophages. By contrast, the GSH concentration in tumors with HIF-1*α* KO macrophages eventually decays to 0.0048 M in a linear fashion over the same period of time. In Figs. 5(c) – (d), we notice that the model did not reproduce the sudden increases of GSH concentration occurring between day 20 and 27 as indicated in the experiments. This suggests that there is an additional latent mechanism for the GSH concentration growth.

**Figure 6:**
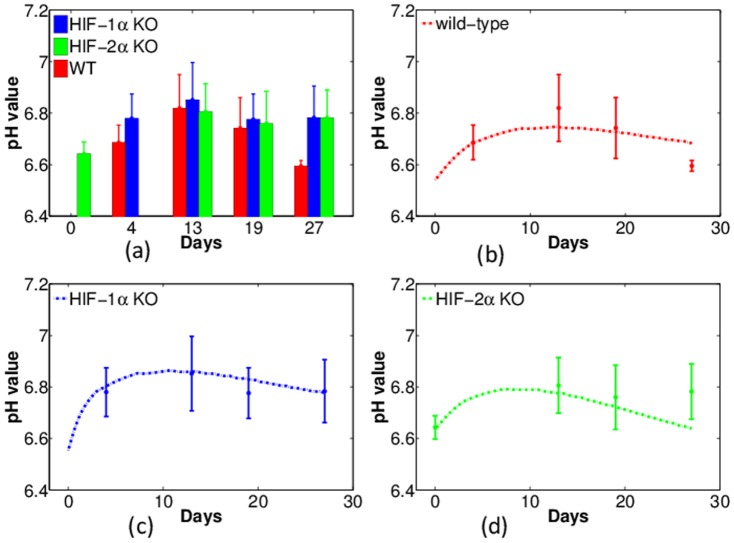
Experiments and simulations of pH in tumors with wild-type, HIF-1*α*-, and HIF-2*α*-deficient macrophages (WT, HIF-1*α* KO, and HIF-2*α* KO). Horizontal axis represents time (in days) and vertical axis shows the pH value. (a): Experimental data of pH against time with error bars. Red: WT; Blue: HIF-1*α* KO; Green: HIF-2*α* KO. (b) – (d): Comparison of experiments (dots with error bars) and numerical simulations (dash curves) of pH in tumor with WT, HIF1-*α*, and HIF-2*α* KO macrophages, respectively.

Tumors usually have a more acidic environment (a lower pH*_e_*) than normal tissue and the pH*_e_* is elevated in the GM-CSF treated tumors [26]. Accordingly, we hypothesize that acidosis will be relieved in tumors with HIF-1*α* KO macrophages, although there could be other factors contributing to the pH when tumor microenvironment is altered. Figure 6 (a) shows the experimental results regarding the level of pH*_e_*: the level is 6.8 in tumors with HIF-1*α* KO macrophages, compared with of 6.6 in tumors with WT macrophages. Surprisingly, as indicated in the figure, the pH*_e_* in tumors with HIF-2*α* KO macrophages is also raised up to a similar level as in tumors with HIF-1*α* KO macrophages.

Part of these features are captured in the model simulations: the simulated pH*_e_* in tumors with WT macrophages is generally below 6.8 (Fig. 6 (b)) and it is elevated above this number in tumors with HIF-1*α* KO macrophages (Fig. 6 (c)). However, the simulations underestimate the pH*_e_* of tumors with HIF-2*α* KO macrophages (or over-estimate the 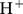 concentration), as seen in Fig. 6(d). The reason could be that we have only taken into account the impact of HIFs on cancer cells while other cells could also contribute to the concentration of hydrogen ions. It is interesting to notice that, in Fig. 6(a), the experimental data of the pH*_e_* level for tumors with the three types of macrophages, all peak on day 13. This feature is also observed in our corresponding simulations in Figure 6 (b) – (d), although the peak values shift to around day 10.

**Figure 7:**
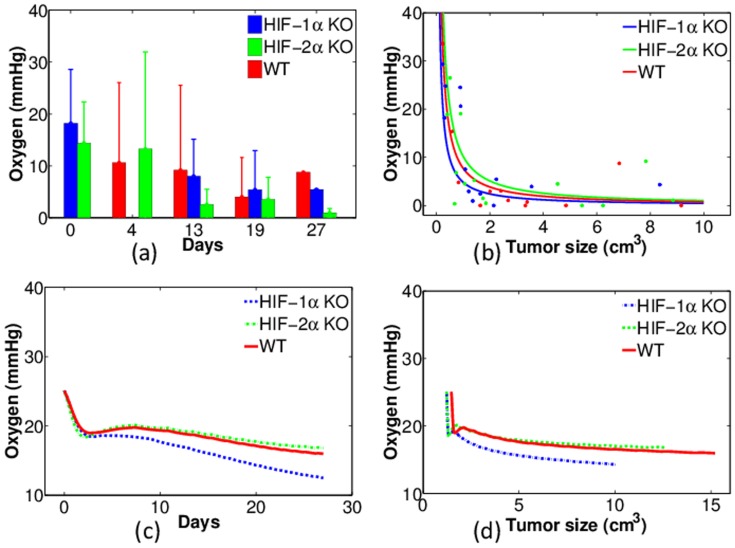
Experiments and simulations of oxygen tension of tumors with wild-type, HIF-1*α*- and HIF-2*α*-deficient macrophages (WT, HIF-1*α* KO and HIF-2*α* KO). (a): Experimental data of oxygen tension (mmHg) against time (days). Red: WT; Blue: HIF-1*α* KO; Green: HIF-2*α* KO; (b): Same experiments aligned with tumor volumes (dots) and the correspondingly fitted curves; (c): Numerical simulations of oxygen tension against time; (d): Numerical simulations of oxygen tension aligned with tumor volumes.

Figure 7 displays the experiments and model simulations of oxygen tension (in units of mmHg). The experimental data of averaged oxygen level taken at several time points are shown in Fig. 7 (a). Since there are relatively large variations among the individual mice, it is difficult to draw conclusions about the impact of HIF-1*α* or HIF-2*α* KO on oxygen tension that is independent of the tumor volume. We therefore proceed from another perspective, to represent the experimental data for the individual mice instead of taking the average. In Fig. 7 (b) the oxygen level is plotted against tumor volume. For better comparison, weighted nonlinear squares fitting was applied (with the reciprocal of experimental variance as weights) to obtain the colored curves fitting to the corresponding dots for each group. Fig. 7(b) suggests that tumors with HIF-1*α* KO macrophages generally have lower oxygen levels than in WT and in HIF-2*α* KO macrophages. By contrast, tumors with HIF-2*α* KO macrophages have higher oxygen levels; this is consistent with the conclusions in [42], and the model simulations in Figs. 7(c) – (d). qualitatively agree with this conclusion.

### The GSH-ROS axis

Intracellular dynamics between ROS and GSH have significant impact on cell's life-cycle, signaling processes, and tumor angiogenesis. Thus, ROS-mediated mechanisms could be used to devise strategies to interfere with the life-cycle of cancer cells in order to inhibit tumor growth. ROS level can be regulated by GSH concentration. In [45], L-Buthionine (BSO) treatment was utilized in a human B lymphoma cell line to achieve intracellular GSH depletion. As a consequence, ROS level was increased and a variety of apoptotic signals of cancer cells were induced even when there were no external apoptotic stimuli. In the current work, we use our model to perform simulations on the effects of GSH depletion in tumor growth.

**Figure 8:**
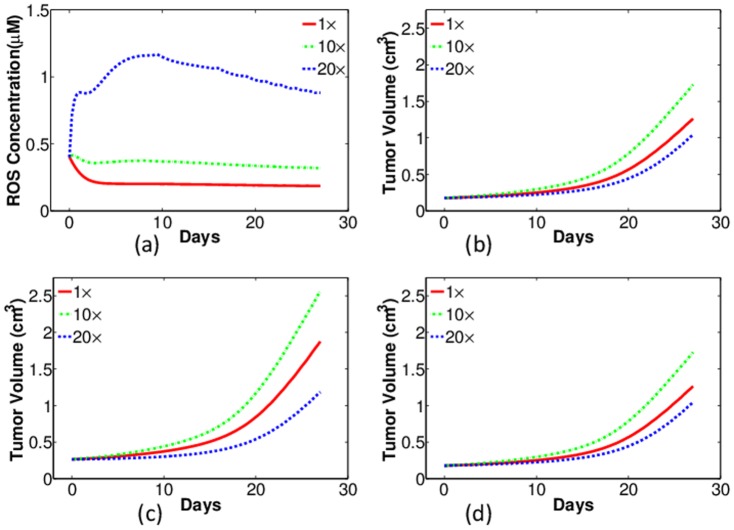
Simulations of intracellular ROS concentration (first row) and tumor growth (second row) with different levels of GSH depletion in tumors with wild-type macrophages (left column) and HIF-1*α* KO macrophages (right column). Red: no depletion (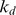); Green: moderate depletion (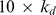); Blue: severe depletion (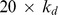). (a–b): ROS levels (*µ*M) against time (days); (c–d): the corresponding tumor volume (cm^3^).

Figure 8 displays the results of regulating intracellular GSH concentration in tumors with WT macrophages 8(a), 8 (c) and HIF-1*α*-deficient macrophages 8(b), 8(d). GSH depletion is simulated by augmenting the GSH degradation coefficient 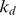 in Eq. (19) to different extents. The red, green, and blue curves are results with no depletion (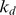), moderate depletion (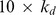), and severe depletion (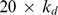), respectively. Fig. 8(a) and 8(b) show the intracellular ROS concentrations in case of WT- and HIF-1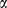-deficient macrophages, respectively. In both cases, when 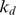 is increased 10 fold, the ROS levels are elevated but still remain below the assumed toxic threshold (0.7 µM), as indicated by the green curves in Figs. 8(a) and 8 (b). Consequently, the corresponding tumor growth, shown by the green curves in Fig. 8(c) and 8(d) are actually promoted, because ROS at this level helps cancer proliferation. By contrast, as shown by the blue curves in the figure, when 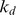 is increased by 20 fold, the ROS levels are elevated above the toxic threshold, and then they damages cancer cells. As a consequence, the tumor growth is suppressed.

By carefully comparing the simulation results in Fig 8 (a) and 8 (b), we notice that the ROS level in tumors with HIF-1*α*-deficient macrophages is slightly less than that in tumors with WT macrophages. This seems to be contradictory to our previous simulations that with HIF-1*α* KO macrophages, GSH concentration in cancer cells is reduced and hence the ROS level is supposed to increase. This apparent contradiction can be explained by the assumption made in the model that ROS production is oxygen level dependent (first term of the right hand side of Eq. (18)): since there is less oxygen in tumors with HIF-1*α* KO macrophages, ROS production is actually reduced in cancer cells.

The therapeutic strategy of GSH depletion is to selectively raise ROS level above the toxic threshold in cancer cells; however, the model indicates that HIF-1*α* knockout in macrophages could reduce intracellular ROS production in tumor cells. Thus, by GSH depletion, tumor volume reduction with HIF-1*α* KO macrophages may be less significant than in tumors with WT macrophages. As shown in Fig. 8 (c), severe depletion of GSH reduces tumor volume from 1.875 cm^3^ to 1.183 cm^3^ on day 27, or a 37% reduction; on the other hand, in tumors with HIF-1*α* KO macrophages, as indicated by Fig. 8 (d), the same amount of GSH depletion reduces the tumor volume from 1.260 cm^3^ to 1.043 cm^3^, or a 17% reduction.

**Figure 9:**
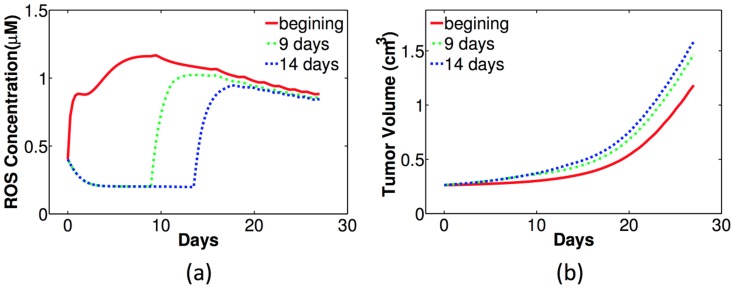
Simulations of intracellular ROS concentration and tumor growth with severe GSH depletion (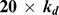) at different time in tumors with wild-type macrophages. Red: GSH depletion at the beginning; Green: GSH depletion starts from the ninth day; Blue: GSH depletion from the fourteenth day. (a) ROS levels (*µ*M) against time (days); (b) the corresponding tumor volume (cm^3^).

In the above simulations, the treatment of GSH depletion was assumed to start at the beginning of tumor growth. But we also simulated the effects of GSH depletion (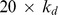) starting at different times of tumor growth. In Figure 9 (a), the ROS levels with GSH depletion starting on the first, the ninth, and the fourteenth day of tumor growth are presented in red, green and blue curves, respectively. Fig. 9 (b) shows the corresponding tumor volumes with these treatments. We see that earlier treatment of GSH depletion will maintain the ROS level above the toxicity threshold for a longer time, and thus has a better effect in suppressing tumor growth.

### Effectiveness of docetacxel treatment

HIFs can regulate tumor microenvironment including GSH concentration, pH, and oxygen tension. Since changes in the tumor microenvironment can have significant impact on both tumor growth and efficacy of chemotherapies, another set of experiments was performed to determine the effectiveness of docetaxel (DTX) chemotherapy for tumors with HIF-1*α*- and HIF-2*α*-deficient macrophages.

**Figure 10:**
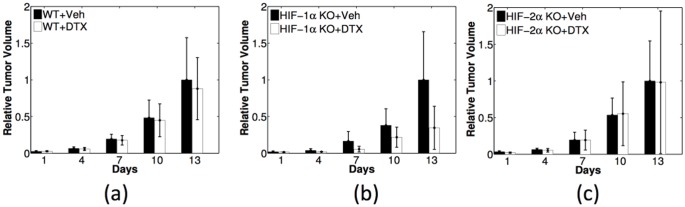
Experiments of testing DTX efficacy in tumors with wild-type, HIF-1*α*-, and HIF-2*α*-deficient macrophages (WT, HIF-1*α* KO and HIF-2*α* KO), in (a)-(c), respectively. Black: non-treated tumors (Veh); white: DTX-treated tumors. Relative tumor volume is obtained by dividing the volume of the treated tumor by the volume of non-treated tumor at the last day of each case.

Figure 10 shows the experiments of non-treated (black bars) and DTX-treated tumor growth (white bars), with WT, HIF-1*α* KO and HIF-2*α* KO macrophages in 10(a)- 10(c), respectively; the black columns of day 13 is normalized by one, and the white columns correspond to tumor volume relative to non-treated tumor. Comparing the black and white bars, we conclude that tumor environment with HIF-1*α* KO macrophages are responding better to the DTX-treatment: tumor volume is reduced to less than 40% of the non-treated tumor, as seen in Fig. 10(b). By contrast, Fig. 10(a) shows that the DTX-treatment has very limited effects (tumor volume is reduced by less than 10%) for tumors with WT macrophages. DTX seems to have no effect on tumors with HIF-2*α* KO macrophages, as shown in Fig. 10(c).

**Figure 11:**
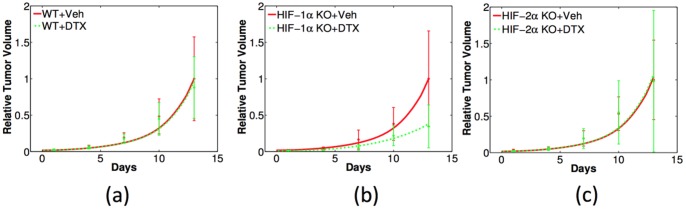
Comparison of simulations (colored curves) with the experiments from **Figure 10** (dots with error bars) for DTX effectiveness in tumors with wild-type, HIF-1*α*-, and HIF-2*α*-deficient macrophages (WT, HIF-1*α* KO and HIF-2*α* KO) in (a) – (c), respectively. Tumor volumes are normalized in the same way as in Figure 10.

Our model can be used to simulate tumor growth with DTX treatment and predict the corresponding characteristics of tumor microenvironment which were not monitored in the above experiments. But before we perform the simulations we need to modify the model in order to incorporate the effect of DTX-treatment. It is known that DTX increases the apoptotic rate of cancer cells by binding to microtubules during mitosis. It is also known [42], [46] that the efficacy of the drug depends on the level of oxygen. Accordingly, we take in Eq. (1) a modified apoptotic rate:
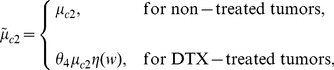
(27)where
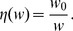
(28)and 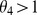. Figure 11 shows that with the choice of 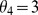 the model simulations are in good fit with the experimental results in Fig. 10. Note that a different set of mice were used in the experiments recorded in Fig. 10 from those in the previous experiments. Hence our simulations in the non-treated case correspond to the mice in Fig. 10, not in Fig. 4.

**Figure 12:**
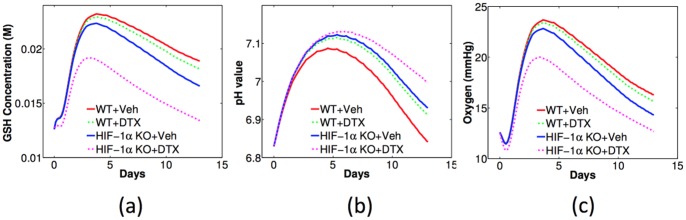
Model simulations of intracellular GSH concentration (a), pH (b), and oxygen tension (c) changing with time in DTX-treated and non-treated tumors, combined with WT or HIF-1*α* KO macrophages.

We can now use the model to predict the change of tumor microenvironment associated with the DTX treatment. Figure 12 shows the model simulations of GSH concentration, pH, and oxygen tension in (a) – (c), respectively. Each panel displays the effect of the combination of DTX treatment and HIF-1*α* knockout. The red and blue solid curves are for non-treated tumor with WT and HIF-1*α* KO macrophages, respectively; the green and magenta dashed curves are for the corresponding tumor with the DTX treatment. Comparing the blue and green curves, we conclude that HIF-1*α* KO in macrophages significantly lowers GSH concentration and reduces oxygen tension in tumor microenvironment than DTX treatment does. Recalling Fig. 10 (b) or Fig. 11 (b), we see that there is a correlation between the effectiveness of DTX and reduced levels of GSH concentration, increased pH, and reduced oxygen tension.

**Figure 13:**
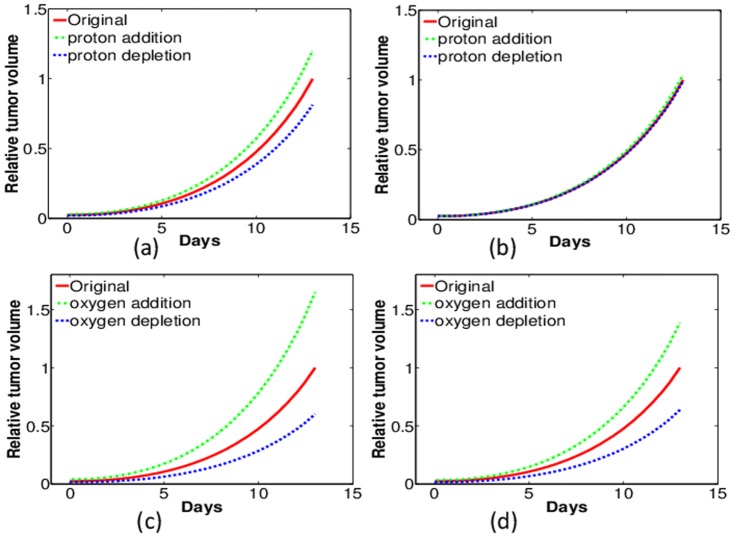
Model simulations of tumor growth with DTX treatment and parameter variations. (a) Proton variation with WT macrophages; (b) Proton variation with HIF-1*α* KO macrophages; (c) oxygen variation with WT macrophages; (d) oxygen variation with HIF-1*α* KO macrophages. The tumor volume without pH or oxygen variation on the last day is normalized to one.

Figure 13 shows the simulated change of tumor growth with DTX treatment and the parameter variations. For clear comparison, the simulation with the same parameters as in Figs. 11 and 12 are in red curves, and the tumor volume on the last day is normalized by one. In these simulations, the parameter 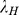 in Eq. (13) is increased by three times (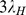) to approximate the “proton addition” and the resulting tumor growth curves are in green, while the parameter 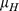 is increased to 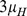 to simulate “proton depletion’ and the corresponding tumor growth is in blue. Fig. 13 (a) and (b) are for pH variations with WT and HIF-1*α* macrophages, respectively. We conclude from the simulations that proton addition (or pH lowering) will reduce the DTX efficacy while proton deletion (or pH enhancing) will increase the efficacy of DTX. These phenomena are enhanced in tumors with WT macrophages than in tumors with HIF-1*α*-deficient macrophages.

Figure. 13 (c) and (d) are for oxygen variations with WT and HIF-1*α* macrophages, respectively. As before, the result with the same parameters as in Figs. 11 and 12 are shown in red curves and the volume on the last day is normalized by one. In these simulations, the parameter 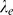 in Eq. (12) is increased to 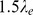 and reduced to 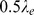 for the “oxygen addition” and “oxygen depletion”, respectively. We conclude that DTX is more effective with lower oxygen tension, while the efficacy of DTX shows no obvious differences in tumors with WT and HIF-1*α*-deficient macrophages.

### Modeling enhanced therapeutic effectiveness

The power of mathematical modeling lies in the ability to alter variables that can be difficult or impossible to manipulate through experimentation and predict changes in outcome to the system. Such predictions are increasingly more valuable when the model system has been validated and correspond to data collected from *in vitro* or *in vivo* experimentation. Using modeling predictions generated from experiments performed on PyMT breast tumors in mice with wild type macrophages or mice with macrophages deficient in either HIF-1*α* or HIF-2*α*, we set out to predict enhanced therapeutic effectiveness to inhibit breast tumor growth based on changes in tumor intracellular glutathione, tumor pH, and tumor oxygen tension in the presence of the chemotherapy agent, docetaxel.

### Summary of model validation by experimental data

- 1) Tumors with macrophages deficient in HIF-1*α* grow slower than tumors with wild type macrophages (Fig. 4).
- 2) Tumors with macrophages deficient in HIF-1*α* have reduced levels of intracellular GSH while tumors with wild type macrophages maintain higher intracellular GSH levels (Fig. 5).
- 3) Tumors with wild type macrophages have a reduced pH compared to tumors with HIF-1*α*- or HIF-2*α*-deficiency (Fig. 6).
- 4) Tumors with HIF-1*α*-deficient macrophages have less average oxygen than tumors with wild type macrophages (Fig. 7).
- 5) Docetaxel is markedly more effective in reducing tumor growth rates in tumors with HIF-1*α*-deficient macrophages than tumors from either wild type or HIF-2*α*-deficient macrophages (Fig. 11).

### Summary of model predictions

- 1) Depleting tumor intracellular GSH by 10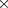 enhances tumor growth in tumors containing either wild type macrophages or HIF-1*α*-deficient macrophages. To the contrary, depleting GSH 20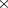 inhibits tumor growth rates in tumors with wild type macrophages but has little or no effect on tumors with HIF-1*α*-deficient macrophages (Fig. 8).
- 2) Depleting tumor intracellular GSH starting at treatment day 1 maximally enhances free ROS leading to slower tumor growth rates in tumors with wild type macrophages, but does not have such an effect on macrophages deficient in HIF-1*α*, most likely because GSH levels in tumors with HIF-1*α*-deficient macrophages are already depleted (Fig. 9).
- 3) Changing tumor pH with DTX treatment alters tumor growth rates more in tumors with wild type macrophages than in tumors with HIF-1*α*-deficient macrophages (Fig. 13) while adding or reducing oxygen with DTX treatment had no differential effect on tumors with wild type macrophages or those tumors with macrophage HIF-1*α*-deficiency (Fig. B(c)(d)).

Our modeling alleges a major contributor to docetaxel effectiveness in inhibiting tumor growth is linked to HIF-1*α*-deficient macrophage regulation of intracellular tumor GSH levels. Studies are underway in our laboratory demonstrating that tumor cells co-cultured with HIF-1*α*-deficient macrophages regulate the expression of tumor cell GSH-building enzymes. Indeed, studies have reported that increased tumor cell GSH levels and overexpression of GSH-synthesizing enzymes both predict a poor prognosis [47] and lead to reduced sensitivity to chemotherapy [48]–[54]. Glutathione is not translated as most other proteins; it is a tripeptide synthesized from the amino acids L-cysteine, L-glutamic acid, and glycine and made in two ATP-dependent steps: First, 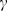-glutamylcysteine is synthesized from L-glutamate and cysteine by the enzyme 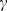-glutamylcysteine synthetase. Second, glycine is added to 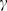-glutamylcysteine by the enzyme glutathione synthetase. Downregulation of these key GSH-building enzymes, along with membrane transporters like 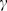-glutamyl transferase in tumor cells, restrict their ability to compensate for ROS build-up, thus making them more susceptible to high ROS as well as limiting their ability to neutralize chemotherapy drugs like docetaxel by GSH. Our study suggests that therapies directed at promoting tumor cell apoptosis, as do most standard chemotherapy compounds, would be greatly enhanced in combination with a small molecule inhibitor specific for macrophage HIF-1*α*. Unexpectedly, because tumors with macrophages deficient in HIF-1*α* display reduced average oxygen tension, our modeling predicts that a similar treatment strategy would be ineffective for ROS-generated killing treatments such as radiation therapy which requires oxygen.

## Conclusions

Tumor growth and effectiveness of chemotherapies greatly depend on the chemical tumor microenvironment. Thus, development of approaches, experimentally and numerically, to study dynamical changes in the tumor microenvironment may provide a key tool for anti-cancer drugs screening and optimization of anticancer therapies. In this work, we focused on several parameters which determine the chemical tumor microenvironment including GSH concentration, pH level and oxygen tension. The use of L-Band electron paramagnetic resonance (EPR) technology and probes developed specifically for each parameter allow for *in vivo*, real-time longitudinal analysis of mouse models of breast cancer. In this model, compared to normal mammary gland tissue, solid tumors generally have lower oxygen tension, lower extracellular pH, and higher intracellular GSH concentration, emulating the environmental parameters of human cancers. Interestingly, we found that this tumor microenvironment can also be altered by the absence or presence of macrophage HIF-1*α* or HIF-2*α*. Experiments had been performed to measure changes in GSH concentration, pH level and oxygen tension as their associated tumors progressed. Concomitantly, experiments were carried out to investigate the effectiveness of docetaxel treatment on tumors with wild-type, HIF-1*α*- and HIF-2*α*-deficient macrophages. In this paper we developed a mathematical model that simulates tumor growth along with the dynamics of GSH concentration, pH, and oxygen tension and how these parameters are altered by the macrophage HIF subunits. The model is multi-scale: interactions among cancer cells, immune system, endothelial cells, oxygen level, hydrogen ions, and corresponding cytokines were described at the tissue level by a coupled system of partial differential equations with a moving boundary, while chemical dynamics among GSH, ROS and other molecules are modeled by a set of ordinary differential equations at the cellular level. The model was validated by the comparison of simulations with experimental data from the prospective of intracellular GSH, pH, and oxygen tension in tumors grown in wild-type (LysMcre), HIF-1*α*-deficient (LysMcre/HIF-1*α^flox/flox^*) and HIF-2*α*-deficient (LysMcre/HIF-2*α^flox/flox^*) mice. Next the model was extended to include treatment with docetaxel (DTX), a chemotherapeutic drug that inhibits disassembly of microtubules during mitotic cell division thus initiating apoptosis. The model for the case of DTX treatment was validated by comparing the simulation with experimental results for tumor growth under DTX treatment, with or without macrophage HIF-1*α* or HIF-2*α*. Clinical trials involving therapeutic manipulation of tumor cell GSH, GSH-building enzymes, and targeting of transcription factors inhibiting these mechanisms are abundant (reviewed extensively in [55]). But our experimental and modeling data demonstrates that contribution of the tumor microenvironment, specifically from tumor macrophages, in the regulation of tumor cell GSH should be considered. Our model suggests an intriguing possibility that tumor-associated macrophages, specifically through HIF-1*α* activity, can augment tumor intracellular GSH to help tumor cells develop a resistance to therapy. Our experimental data and modeling predictions were obtained using the PyMT orthotopic breast tumor implantation model to understand the role of HIF transcription factors in regulating the chemical tumor microenvironment and a consequence on chemotherapy effectiveness. It would be interesting to perform similar longitudinal experiments tracking tumor GSH, pH, and oxygen in transgenic PyMT mice with wild type macrophages which spontaneously form mammary tumors starting at 4 weeks of age and progress through all four stages similar to human breast cancer [56] to understand the changes in these parameters as the tumor progresses to malignancy.
